# *In Vitro* and *In Vivo* Development of Horse Cloned Embryos Generated with iPSCs, Mesenchymal Stromal Cells and Fetal or Adult Fibroblasts as Nuclear Donors

**DOI:** 10.1371/journal.pone.0164049

**Published:** 2016-10-12

**Authors:** Ramiro Olivera, Lucia Natalia Moro, Roberto Jordan, Carlos Luzzani, Santiago Miriuka, Martin Radrizzani, F. Xavier Donadeu, Gabriel Vichera

**Affiliations:** 1 KHEIRON S.A Laboratory, Pilar, Buenos Aires, Argentina; 2 Laboratory of Biology of Cell Development, LIAN-Unit associated with CONICET, FLENI, Belen de Escobar, Buenos Aires, Argentina; 3 Laboratory of Neruogenetic and Molecular Cytogentic, School of Sciences, National University of San Martin, CONICET, Buenos Aires, Argentina; 4 The Roslin Institute and Royal School of Veterinary Studies, University of Edinburgh, Easter Bush, Midlothian, United Kingdom; 5 Euan MacDonald Centre for MND Research, University of Edinburgh, Edinburgh, United Kingdom; Michigan State University, UNITED STATES

## Abstract

The demand for equine cloning as a tool to preserve high genetic value is growing worldwide; however, nuclear transfer efficiency is still very low. To address this issue, we first evaluated the effects of time from cell fusion to activation (<1h, n = 1261; 1-2h, n = 1773; 2-3h, n = 1647) on *in vitro* and *in vivo* development of equine embryos generated by cloning. Then, we evaluated the effects of using different nuclear donor cell types in two successive experiments: I) induced pluripotent stem cells (iPSCs) *vs*. adult fibroblasts (AF) fused to ooplasts injected with the pluripotency-inducing genes *OCT4*, *SOX2*, *MYC* and *KLF4*, *vs*. AF alone as controls; II) umbilical cord-derived mesenchymal stromal cells (UC-MSCs) *vs*. fetal fibroblasts derived from an unborn cloned foetus (FF) *vs*. AF from the original individual. In the first experiment, both blastocyst production and pregnancy rates were higher in the 2-3h group (11.5% and 9.5%, respectively), respect to <1h (5.2% and 2%, respectively) and 1-2h (5.6% and 4.7%, respectively) groups (P<0.05). However, percentages of born foals/pregnancies were similar when intervals of 2-3h (35.2%) or 1-2h (35.7%) were used. In contrast to AF, the iPSCs did not generate any blastocyst-stage embryos. Moreover, injection of oocytes with the pluripotency-inducing genes did not improve blastocyst production nor pregnancy rates respect to AF controls. Finally, higher blastocyst production was obtained using UC-MSC (15.6%) than using FF (8.9%) or AF (9.3%), (P<0.05). Despite pregnancy rates were similar for these 3 groups (17.6%, 18.2% and 22%, respectively), viable foals (two) were obtained only by using FF. In summary, optimum blastocyst production rates can be obtained using a 2-3h interval between cell fusion and activation as well as using UC-MSCs as nuclear donors. Moreover, FF line can improve the efficiency of an inefficient AF line. Overall, 24 healthy foals were obtained from a total of 29 born foals.

## Introduction

Cloning is currently the only proven technique to replicate valuable animals.

In the horse, the use of cloning can be justified for the large number of extremely valuable individuals worldwide and an increasing demand to multiply them. However, the limited number of available horse oocytes and the complexity of cloning necessitate significant technical improvement to enhance efficiency and reduce associated costs. Most studies have reported low blastocyst and high pregnancy loss after somatic cell nuclear transfer (SCNT), with usually ≤ 5% of transferred embryos resulting in healthy foals [1, 2, 3, 4, 5, 6, 7]. On the other hand, few reports have informed high blastocysts and live foals rates [8, 9, 10].

The low efficiency and disparity of the results among groups is related to the highly complex process that the cloning technique requires. For cloning to be successful, the differentiated state of the donor cell needs to be reset to an embryonic state within a relatively short period of time between embryo reconstruction and activation [11, 12]. Some reports have focused on this step, which has demonstrated to be beneficial to epigenetic reprogramming and normal embryo development in different mammalian species [13, 14, 15]. However, when this interval was prolonged embryo development was compromised [16, 17, 18], probably due to oocyte aging [19].

To improve horse cloning efficiency different alternatives have been reported. Roscovitine was used to induce G1 phase arrest in donor cells, making them more susceptible to reprogramming by the oocyte cytoplasm [9, 20]. Moreover, embryo aggregation of 2, 3 or 4 zona-free cloned embryos has been tested in order to compensate embryo-autonomous reprogramming and improve developmental competence [5, 6]. Although few born foals were obtained using both roscovitine treatment and embryo aggregation, they observed an increase in the proportion of viable offspring born after SCNT [5, 6, 9].

In addition, the nuclear donor cell type also influences over the capacity of achieving an undifferentiated state after nuclear reprogramming. Using foetal fibroblasts (FFs) rather than adult fibroblasts (AFs) in the horse led to an increase in blastocyst rates [4]. In several species pluripotent and multi-potent stem cells have been used as nuclear donors. In pigs, full-term development of cloned embryos was significantly higher using mesenchymal stem cells (MSCs) rather than AFs [21]. Moreover, the induced pluripotent stem cells (iPSCs) have emerged as an alternative to SCNT through which nuclear reprogramming can be achieved *in vitro* with relative high efficiency [22]. Nuclear transfer has been performed in mice using iPSCs as nuclear donors [23], which provided relatively high efficiency and resulted in viable offspring. The same procedure was evaluated in pigs but failed to generate offspring [24], presumably due to the persistent expression of the reprogramming transgenes in the donor iPSCs [24, 25]. Although horse MSCs have been isolated from many tissues [26, 27, 28, 29, 30, 31, 32] and horse iPSCs have been already generated [33, 34, 35], their potential as donor cells for cloning has not been evaluated yet.

In the present study, we determined the *in vitro* and *in vivo* efficiency of horse cloning evaluating different time periods between cell fusion and activation using AFs as nuclear donors. Moreover, we evaluated the effects of microinjecting the reprogramming genes used to generate the iPSCs (*OCT4*-*KLF4*-*MYC*-*SOX2*) to assist the nuclear transfer procedure in the horse, and different cell types as nuclear donors, including iPSCs, umbilical cord MSCs (UC-MSCs), FFs or AFs.

## Materials and Methods

### Reagents

Except when otherwise indicated, all chemicals were obtained from Sigma Chemical Company (St. Louis, MO, USA).

### Care and Use of Research Animals

This study was carried out according to the guidelines stated in the Guide for the Care and Use of Agricultural Animals in Agricultural Research and Teaching. The protocol was approved by the Institutional Committee for the Care and Use of Experimental Animals of the San Martin National University, Buenos Aires, Argentina (CICUAE-UNSAM, Permit Number: 001/16). All procedures were performed by qualified veterinarians and all efforts were made to minimize animal suffering or stress. Animal health (both mares and foetuses) were monitored once a week.

### Oocyte collection and in vitro maturation

Horse ovaries were collected from three different slaughterhouses (Lamar S.A., ruta 5 km 94, Mercedes, Buenos Aires, Argentina, zip code: B6600; Raul Aimar S.A., ruta 36 km 597, Rio Cuarto, Córdoba, Argentina, zip code: 5805; and E. Rios S.A., calle pública SN, Gualeguay, Entre Rios, Argentina, zip code: 2840) and transported to the laboratory in a thermic bag at 25–29°C within 2–4 h after slaughter. Fluid from visible follicles was removed using an 18-G needle and a 10 ml syringe. Follicles were then opened with a scalpel blade and the cumulus-oocyte complexes (COCs) were recovered by a combination of scraping and washing the follicular walls with Dulbecco´s modified Eagle Medium (DMEM, 11885, Gibco, Grand Island, NY, USA) supplemented with 1 mM sodium pyruvate (P2256), and 15 IU/ml heparin (H3149-50KU). COCs were searched in Petri dishes under a stereomicroscope. For *in vitro* maturation, the COCs were cultured in 100 μl microdroplets containing bicarbonate-buffered TCM-199 (31100–035; Gibco) supplemented with 10% foetal bovine serum (FBS, 10499–044; Gibco), 1 μl/ml insulin-transferrin-selenium (ITS; 51300–044, Gibco), 1 mM sodium pyruvate (P2256), 100 mM cysteamine (M-9768), 0.1 mg/mL of follicle-stimulating hormone (NIH-FSH-P1, Folltropin®; Bioniche, Belleville, ON, Canada) and 2% antibiotic–antimycotic (ATB; penicillin, streptomycin and amphotericin B; 15240–096; Gibco), under mineral oil (M8410) in 5% CO_2_ and humidified air at 39°C, for 22–24 h.

### Removal of cumulus and zona pellucida

After *in vitro* maturation the oocytes were denuded of cumulus cells by pipetting in hyaluronidase solution (H4272, 1 mg⁄ml in Tyrode’s albumin lactate pyruvate medium buffered with HEPES, TALP-H [36]) for 1 min and washed three times in TALP-H. Only those oocytes with a visible first polar body were used. In order to remove the zona pellucida, matured oocytes were incubated in 1.5 mg/ml pronase (P-8811) for 3–8 min at 35°C and returned to the incubator until DNA staining for enucleation.

### Microinjection of pluripotency-inducing genes

**Fig 1:**
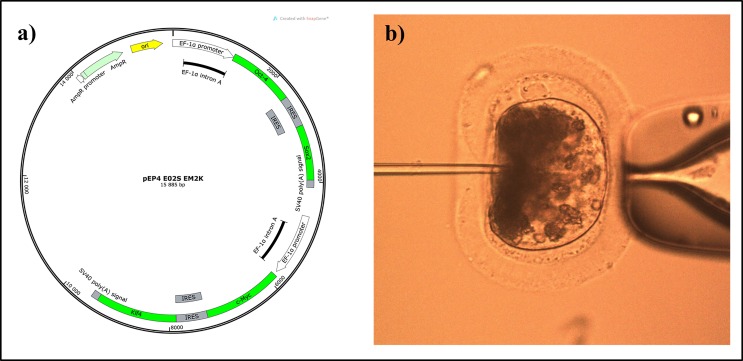
Microinjection of non-integrative pluripotency-inducing genes in horse oocytes before enucleation. a) Plasmid construct containing the *OCT4*, *SOX2*, *MYC* and *KLF4* human sequences. b) Photomicrograph showing the plasmid microinjection procedure.

After the removal of cumulus cells, mature oocytes were microinjected with the pEP4-E02s-EM2k plasmid, which codes for the human genes *OCT4*, *SOX2*, c-*MYC* and *K1F4* sequences (Addgene 20923, Fig 1). The method used for DNA microinjection was previously described by Vichera *et al*. [37] in bovine, and included liposome vesicles for higher transgenesis rates. Briefly, 2 pl of a mixture 1/3 of plasmid/liposomes (FuGENE® 6 Transfection Reagent, Promega, Madison, WI, USA), diluted to half concentration with 10% polyvinylpyrrolidone (PVP, 99219; Irvine Scientific, Santa Ana, CA, USA) were intracytoplasmically injected using an injection capillary of 0.7 μm in diameter (final DNA concentration of 0.5 μg/ml).

### Oocyte enucleation

Prior to enucleation, the zona-free oocytes were incubated with 1 μg/ml Hoechst bisbenzimide 33342 (H33342) for 5 min. A closed holding pipette was used to support the oocyte during enucleation and the metaphase plate was aspirated using a blunt pipette (14 um inner diameter) under UV light, by micromanipulation with a Narishige hydraulic micromanipulator (Medical Systems, Great Neck, NY, USA) mounted on a Nikon Eclipse Ti microscope (Nikon,Melville, NY, USA). Enucleation was confirmed by observing the stained metaphase plate inside the pipette under UV light. Enucleated oocytes were kept in DMEM/F-12 HAM medium (DMEM/F12, D8062) containing 10% FBS and 1% ATB for 15–30 min until fusion.

### Fibroblast Culture

For fibroblast culture, small skin biopsies were obtained from 13 different horses (10 for the first experiment, 1 for the second experiment and 2 for the third experiment). Foetal fibroblasts were produced from the skin sample of a spontaneously aborted 5-month-old foetus cloned from an adult fibroblast cell line used as control group. Fibroblasts were cultured in DMEM with 10% FBS, 1% ATB and 1μl/ml of ITS in 5% CO_2_ in humidified air at 39°C. After 4–7 days, fibroblasts were sub-cultured and expanded at most three times until freezing in DMEM with 20% FBS and 10% DMSO in Mr Frosty^TM^ Freezing Container placed at -80°C for 24 h followed by storage in liquid nitrogen. Quiescence of donor cells was induced by growth to confluence in 0.5% FBS for 2–3 days before nuclear transfer (NT). The cells used for embryo reconstruction were harvested by trypsinization 20 min prior to NT, then washed and re-suspended in the same medium used for culture.

### Mesenchymal umbilical cord cell culture

For UC-MSC isolation a 20 cm portion of an umbilical cord from one animal was collected immediately after birth and placed in 500 ml of PBS with ATB. The sample was processed within 12 h after birth. Briefly, the sample was washed with sterile PBS and dissected to separate blood vessels. Perivascular region of the umbilical cord was then cut into 0.5 cm fragments and placed in 50 ml conical tubes with 15 ml of 1 mg/ml type IV collagenase (17104–019, Invitrogen, CA, USA) at 37°C for 60 min with occasional agitation. Tubes were centrifuged for 5 min at 200 rpm to separate undigested fragments. Supernatant was placed in a new conical tube and centrifuged for 10 min at 1000 rpm. Cells were washed once with PBS, resuspended in DMEM with 10% FBS and plated onto 100 mm dishes at a density of 1×10^4^ cells/cm^2^. When confluent, cells were detached from culture plates and frozen as described previously.

### Multilineage differentiation of MSCs

Multilineage differentiation potential of UC-MSC was assessed using StemPro Adipogenesis (A10070-01, Gibco), Osteogenesis (A10072-01, Gibco) or Chondrogenesis (A10071-01, Gibco) kits, as per manufacturer instructions. After differentiation, adipogenesis induction was assessed by Sudan Black staining. Briefly, cells were fixed with 4% paraformaldehyde solution for 45 min at room temperature, stained with a Sudan Black saturated solution in 70% ethanol for 5 min at room temperature and finally washed thoroughly with 70% ethanol. Osteogenesis induction was assessed by Alzarin Red staining. Fixed cells were stained in a 2% Alzarin Red solution (pH 4.2) for 3 min at room temperature and then washed thoroughly with distilled water. Finally, chondrogenic induction was assessed by Alcian Blue staining. Fixed cells were stained in a 1% Alcian Blue solution prepared in 0.1 N HCl for 30 min at room temperature and then washed thoroughly with distilled water. All three preparations were visualized under light microscope. Images were acquired using a Nikon Eclipse TE2000-S inverted microscope and the Eclipse Net software.

### iPSCs culture

The iPSC-line used in this work was generated by Breton et al. [34]. This iPSC-line was generated by infecting horse fibroblasts with retrovirus containing the Moloney Murine Leukemia Virus backbone plasmid (pMXs) with mouse cDNA sequences for *Oct4*, *Sox2*, *Klf4* and *c-Myc*. One vial of frozen iPSCs colonies was thawed at 37°C 24 h before NT. Cells were cultured in DMEM supplemented with 20% FBS, 2 mM l-glutamine (G8540), 0.1mM β-mercaptoethanol (21985023, Invitrogen), 0.1mM MEM nonessential amino acids (M7145), 1% ATB, 8 ng/ml human basic fibroblast growth factor (bFGF, F0291) and 1,000 IU/ml human leukemia inhibitory factor (LIF, L5283).

### Nuclear transfer and embryo reconstruction

For embryo reconstruction, zona-free ooplasts were individually transferred to a 50 μl drop of 1mg/ml phytohemagglutinin (PHA; L8754) dissolved in TCM-199 for 2–3 s. After that, they were quickly dropped over a single donor cell (fibroblast, MSCs or iPSCs) after which the two structures stuck together. The couplets were placed in fusion medium (0.3 M mannitol, 0.1 mM MgSO4, 0.05 mM CaCl_2_, 1mg/ml polyvinyl alcohol) for 1 min and then moved to a fusion chamber containing 2 ml of fusion medium at 35°C. Membrane fusion was achieved giving a double direct current pulse of 1.2 kV/cm, each pulse for 30 μs, separated by 0.1 s. Twenty min later, fusion was assessed and non-fused couplets were re-fused using the same parameters. After fusion, the zona-free reconstructed embryos were placed individually in 5 μl droplets of DMEM/F12. After that, the reconstructed embryos were activated with 8.7 μM ionomycin (I24222; Invitrogen, Carlsbad, CA, USA) in TALP-H for 4 min followed by individual culture in a combination of 1 mM 6-dimethylaminopurine (6-DMAP; D2629) and 5 mg/ml cycloheximide (CHX; C7698) in 5 μl drops of DMEM/F12, for 4 h.

### Embryo culture

Zona-free reconstructed embryos (ZFRE) were cultured in DMEM/F12 containing 10% FBS, and 1% ATB, in a humidified gas mixture (5% CO_2_, 5% O_2_, 90% N_2_) at 39°C. Embryo aggregation of three ZFRE was performed for all the experimental groups by using the well of the well (WOW) system. The WOW system consists on the generation of microwells in a petri dish, which allows individual or aggregated embryo culture that share culture media among wells. Cleavage was assessed 72 h after activation and half of the medium was renewed at that time. Blastocyst formation was evaluated after 7 and 8 days of culture. The blastocysts were transferred to synchronized mares either at day 7 or 8.

### Embryo transfer, ultrasonographic monitoring and clone birth

Non-surgical embryo transfers to recipients were performed over 3 breeding seasons (2012–2015) in Buenos Aires, Argentina, southern hemisphere, from September to February. All embryos were transferred to recipient mares aged 4 to 10 years old (2 blastocysts per recipient, except when otherwise indicated). To determine the stage of the oestrus cycle each mare was examined 6 days a week by transrectal ultrasonography (Lineal Mindray DP10 ultrasonographer). Ovulations were synchronized by administration of 0.15 mg Prostaglandin F2α (D-Cloprostenol, Emefur, Merial, Buenos Aires, Argentina) and 1500 IU hCG (Ovusyn, Syntex, Buenos Aires, Argentina). Recipients were selected according to ultrasonographic characteristics of uterus and corpus luteum, uterus tone at rectal examination and past reproductive history. Blastocysts were transported at 38°C, in 0.5 ml straw containing DMEM/F12 and transferred within 1 h. Transcervical transfers of day-7 to -8 blastocysts were performed 6–7 days after ovulation using an embryo transfer sheath, after sanitizing the perianal area of the mare, avoiding any pain or stress to the recipient mare. Pregnancies were diagnosed by transrectal ultrasonography 7–15 days after embryo transfer. Mares that were pregnant were treated with 1500 mg Biorelease P4 LA300 (BET Pharm LLC, Lexington, KY, USA) and monitored once a week thereafter. Foetal monitoring consisted of observing foetal movements, heart rate and analysing the quality of amniotic fluid in order to detect foetal suffering at the earliest possible. Between 20 to 30 days before expected parturition, the pregnant mares were transported to an equine hospital (KAWELL, Equine Rehabilitation Centre, Solís, Argentina) to give birth.

### Confirmation of Clones

To confirm that the foals born were indeed clones, 15 loci were compared in genomic DNA between each foal and the respective donor animal (Veterinary Genetics Laboratory, University of California, Davis, CA or Laboratorio de Genética Aplicada Sociedad Rural Argentina, Buenos Aires, Argentina). Each locus was the same between all the cloned foals and the donor animals.

### Statistical analysis

*In vitro* and *in vivo* embryo development endpoints were compared by Chi-square or non-parametric Fisher’s exact test using Statistix version 0.8 software. In all cases, differences were considered significant at P<0.05.

## Results

### In vitro and in vivo development of zona-free aggregated horse embryos subjected to different time periods between cell fusion and activation

**Fig 2:**
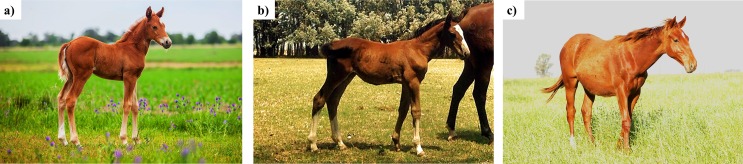
a) Cloned polo pony derived from 1-2h group in experiment 1, born on October 2013, b) Jumping horse cloned derived from 2-3h group in experiment 1, born on November 2014, c) Thoroughbred horse cloned derived from the control group in experiment 2, born on June 2014.

We determined the effects of 3 different time periods (between nuclear transfer and activation), less than 1 h (<1h group), between 1 h and 2 h (1-2h group) and between 2 h and 3 h (2-3h group), on *in vitro* and *in vivo* development of horse clones. Blastocyst development rates were analyzed per embryo and per ZFRE. A significant improvement in blastocyst rates was observed as fusion-to-activation time increased, with the best results obtained in the 2-3h group: 11.5%, 5.6% and 5.2% for 2-3h, 1-2h and <1h groups, respectively (Table 1). We next examined the *in vivo* effects, including pregnancy rates, number of offspring and viable offspring. The results *in vivo* agreed with those obtained *in vitro*, obtaining the highest pregnancy rates (9.5%) in the 2-3h group (Table 1) and the lowest one in the <1 h group (2.0%). In this experiment, a total of 24 foals were born, 19 from the 2-3h group, 5 from the 1-2h group and 0 from the <1 h group. Foals viability differed between groups as 2/5 of the born foals died in the 1-2h group compared to 2/19 in the 2-3h group. Those foals died during birth or immediately after birth, mainly as consequence of umbilical abnormalities (enlargement, omphalocele, schistosomes), observing also limb deformities and failure of passive transfer. These abnormalities are commonly observed in cloned foals [6, 38, 39, 40]. By December 2015, the 20 foals from both groups remain healthy (Fig 2A and 2B).

**Table 1:** Effects of different fusion-to activation times on *in vitro* and *in vivo* development of horse clones.

				Blastocyst production				Pregnancies			Born foals	
Fusion to activation times	No. of ZFRE	No. of embryos (Wells)*	No. of cleaved (%)	No.	% per embryo	% per ZFRE	Transferred embryos (No. recipients)	No.	% per transferred embryo	% per recipient	No. Offspring (%)	No. of viable offspring (%)
2-3h	4941	1647	4348 (88.0)^a^	568	34.5^a^	11.5^a^	568 (284)	54	9.5^a^	19.0^a^	19 (35.2)	17 (31.5)
1-2h	5319	1773	4792 (90.1)^b^	296	16.7^b^	5.6^b^	296 (148)	14	4.7^b^	9.5^b^	5 (35.7)	3 (21.4)
<1h	3783	1261	3029 (80.1)^c^	196	15.5^b^	5.2^b^	196 (98)	4	2.0^b^	4.1^b^	0	-

(a, b, c) Values with different superscripts in a column are significantly different (Fisher’s exact test p<0.05).

ZFRE, zona-free reconstructed embryos.

*Embryo aggregation was performed and each well contained three ZFRE

### Nuclear transfer using iPSCs and injection of pluripotency-inducing genes

We compared the effects of injecting recipient oocytes with pluripotent gene sequences before SCNT (AF+PGI group, for AF+pluripotent genes injection) or using iPSCs as donor cells for SCNT (iPSCs group) on *in vitro* embryo development, pregnancy rates and foal births. Standard SCNT using donor adult fibroblasts (AF) was used as control. Statistical differences in cleavage rates were observed between AF+PGI and AF groups (90.5% and 85.0%, respectively) (Table 2), with no differences in blastocyst rates (12.7% and 9.4%, respectively). However, the embryos generated with the iPSCs had similar cleavage rates as the other two groups (87.0%) but none was able to develop to blastocyst. Pregnancy rates did not differ statistically between the AF+PGI and the AF groups, 3/32 (9.4%) *vs*. 4/43 (9.3%), respectively. The pregnancy from the AF-PGI group resulted in the birth of one foal that died after 72 h as a consequence of umbilical complications and systemic failure. On the other hand, both foals born in the control group were healthy (Fig 2C).

**Table 2:** *In vitro* and *in vivo* development of clone horse embryos reconstructed with iPSCs or after microinjection of recipient oocytes with pluripotent gene sequences.

				Blastocyst production				Pregnancies			Born foals	
Groups	No. of ZFRE	No. of embryos (Wells)*	No. of cleaved (%)	No.	% per embryo	% per ZFRE	Transferred embryos (No. recipients)	No.	% per transferred embryo	% per recipient	No. Offspring (%)	No. of viable offspring (%)
iPSCs	270	90	235 (87.0)^a^^b^	0	0^a^	0^a^	-	-	-	-	-	-
AF+PGI	252	84	228 (90.5)^a^	32	38.1^b^	12.7^b^	32 (16)	3	9.4	18.8	1 (33.3)	0
AF	459	153	390 (85.0)^b^	43	28.1^b^	9.4^b^	43 (21)	4	9.3	19.0	2 (50)	2 (50)

(a, b) Values with different superscripts in a column are significantly different (Fisher’s exact test p<0.05).

ZFRE, zona-free reconstructed embryos; iPSCs, induced pluripotent stem cells; AF, adult fibroblasts; PGI, pluripotent genes injection.

* Embryo aggregation was performed and each well contained three ZFRE

### MSCs and foetal fibroblasts as nuclear donors in zona-free horse cloning

**Fig 3:**
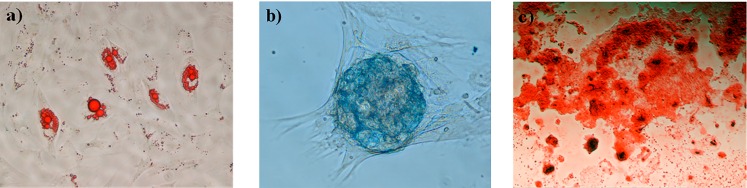
Multilineage differentiation of MSCs. Representative images of MSCs differentiated towards adipogenic (a), chondrogenic (b) or osteogenic (c) lineages.

We isolated MSCs from the umbilical cord of a new-born foal, and we confirmed that they could undergo lineage differentiation towards adipocytes, osteoblast or chondroblast (Fig 3), demonstrating their pluripotent competence. In this experiment, UC-MSCs, FF and AF were used as nuclear donor cells. We obtained higher cleavage rates when UC-MSCs were used (89.8%, 83.3% and 79.9% for UC-MSCs, FF and AF respectively) (Table 3), as well as higher blastocyst rates (15.6%, 8.9% and 9.3% for UC-MSC, FF and AF, respectively). However, this improvement achieved in the *in vitro* development was no reflected in the *in vivo* development. Similar pregnancy rates were observed for all the groups [3/35 (8.6%), 2/22 (9.1%) and 2/19 (10.5%), for UC-MSC, FF and AF, respectively], though only both pregnancies derived from the FF group resulted in 2 healthy clones.

**Table 3:** Effect of the donor-cell differentiation state in *in vitro* and *in vivo* development of horse embryo clones.

				Blastocyst production				Pregnancies			Born foals	
Groups	No. of ZFRE	No. of embryos (Wells)*	No. of cleaved (%)	No.	% per embryo	% per ZFRE	Transferred embryos (No. recipients)	No.	% per transferred embryo	% per recipient	No. Offspring (%)	No. of viable offspring (%)
UC-MSC	225	75	202 (89.8)^a^	35	46.7^a^	15.6^a^	35 (17)	3	8.6	17.6	0	-
FF	246	82	205 (83.3)^b^	22	26.8^b^	8.9^b^	22 (11)	2	9.1	18.2	2 (100)	2 (100)
AF	204	68	163 (79.9)^b^	19	27.9^b^	9.3^a^^b^	19 (9)	2	10.5	22.2	0	-

(a, b) Values with different superscripts in a column are significantly different (Fisher’s exact test p<0.05).

ZFRE, zona-free reconstructed embryos; UC-MSCs, mesenchymal stromal cells isolated from the umbilical cord; FF, foetal fibroblasts; AF, adult fibroblasts

* Embryo aggregation was performed and each well contained three ZFRE.

## Discussion

Although significant progress has been made over the past years, many aspects of horse cloning still need to be optimized to bring about improvements in blastocyst rates and viable offspring [38]. This progress includes the use of roscovitine for synchronization of the donor cell, utilization of sperm extract for oocyte activation [9, 20, 41], fusion with zona-free oocytes [1, 4] and embryo aggregation [5, 6]. In view of this, the experiments reported in this manuscript used zona-free oocytes and embryo aggregation.

In the first experiment, we compared different time periods between cell fusion and oocyte activation. Our results showed an improvement on *in vitro* and *in vivo* embryo development as well as pregnancy rates when the exposure time of the somatic cell nucleus to oocyte cytoplasm was increased up to 3 h. Similar observations were reported for murine NT [42], whereas in bovine longer nuclear fusion-to-activation times were beneficial in some experiments [43, 44] but led to lower *in vitro* blastocyst development in others [45]. In the horse, no differences in cleavage was reported after comparing 30 min and 2 h time windows between fusion to activation, in contrast to our findings [46]. However, Choi et al. [15] have recently demonstrated that the use of horse oocytes immediately after reaching MII, combined with longer time periods (5- or 8-h) from reconstruction to activation, increased developmental competence after cloning. These results contrasts with those reported in the bovine, which determined that too prolonged exposure to arrested MII oocyte cytoplasm may result in a high incidence of structural abnormalities in nuclear material [11]. Our data provides a minimum interval of time required for the donor cell to undergo appropriate embryo development. In this experiment we obtained a total of 24 offspring, adding up to 31.5% of healthy foals per pregnancy when 2–3 h was allowed between fusion and activation. However, future experiments are warranted in order to strengthen these results because fewer embryos were transferred in 1–2 h and <1hs groups as consequence of lower *in vitro* embryo development.

We also evaluated for the first time in the horse the developmental capacity of iPSCs as nuclear donors in cloning, by comparing them with adult fibroblasts fused to ooplast that had been injected with pluripotency-inducing gene sequences typically used to generate iPSCs. Horse iPSCs have been produced by only few groups [33, 34, 47]. In this study, we used a cell line previously generated by us [34], capable of differentiating into derivatives of the three germ layers both *in vitro* and *in vivo*. We found that reconstructed embryos from iPSCs cleaved normally but blocked without undergoing blastocyst formation. In another study, embryos derived from 5 different pig iPSC lines failed to develop to term after transfer [24] presumably because of the persistent expression of the pluripotency-inducing genes. Failure to silence the reprogramming transgene is a common feature of iPSCs from domestic species including bovine [48], swine [24, 49, 50] and ovine [51]. Moreover, another report in ovine obtained very low blastocyst development rates using iPSCs as nuclear donors and found that nuclear transfer blastocysts produced with fibroblasts were better reprogrammed [52]. However, cloned mice have been generated using iPSCs [23] demonstrating the capacity of these cells to become totipotent by nuclear transfer. It is conceivable then that the reported persistent expression of *OCT4* by our horse iPSCs [34] and by iPSCs from other species generated using the same reprogramming system [53, 54, 55], was a main cause of cloning failure. Despite our results, we do not rule out that iPSCs generated using non-integrating vectors could be efficient nuclear donors and generate viable offspring in the horse. In fact, we explored this possibility by injecting episomal vectors that encode for *OCT4*, *SOX2*, *MYC* and *K1F4* in the cytoplasm of recipient oocytes before fusion. We observed an increment of blastocyst production rates (12.7%) using injected oocytes. Now, it is necessary to explore the molecular mechanisms by which high expression of *OCT4*, *SOX2*, *MYC* and *K1F4* improves reprogramming of the donor nucleus in conventional cloning.

We also compared the capacity of UC-MSCs and FFs to develop *in vitro* and *in vivo* after nuclear transfer. To our knowledge, there are no reports using MSCs as nuclear donors in horse cloning, but there are some in other species [56, 57, 58, 59, 60, 61]. One healthy clone derived from adult MSCs in the bovine was generated before, however offspring production rates were not better than those obtained with other somatic cell types [62], consistent with another report [63]. In our study, significant higher blastocyst rates were obtained using UC-MSCs than FFs or AFs but this trend was not maintained after embryo transfer, as pregnancy rates were similar among the 3 groups and no offspring was born using UC-MSCs. In contrast to UC-MSCs, *in vitro* and *in vivo* embryo development using FFs as nuclear donors were similar to using AFs. However, we obtained healthy foals only from the FF group in this experiment. In a previous report, FFs have been used for horse cloning with good results in terms of blastocyst rates but not after transfer into recipients [4], in contrast to our findings. We consider that the fibroblast line used was inefficient in terms of embryo developmental capacity and we enhance its potential by using fetal cells derived from an aborted foetus from the inefficient line. With these results, we propose this alternative with problematic lines in order to enhance their potential.

Overall, we obtained 29 offspring of which 24 remain healthy. The abnormalities detected in the 5 foals that died after birth were common findings in foals derived using the cloning [39].

## Conclusions

In summary, this is to our knowledge the first report that compares different donor cell types encompassing a wide range of developmental potentials for horse cloning, resulting in a larger number of reconstructed embryos and born foals than in any previous study, which strengthens our conclusions. Our results identified a minimal timeframe required between fusion and activation leading to successful cloning, as no pregnancies were obtained when <1 h was allowed. Moreover, donor iPSCs did not produce any blastocyst; however, based in our results using injection of pluripotency-inducing genes into recipient oocytes, the potential of integration-free horse iPSCs as donor cells for cloning should be explored in the future. Finally, UC-MSCs demonstrated high developmental potential *in vitro* and FFs turned out to be the most appropriate cell source to be used as nuclear donor in terms of *in vivo* development to term in horse cloning.
